# Insights Into the Sunlight-Driven Water Oxidation by Ce and Er-Doped ZrO_*2*_

**DOI:** 10.3389/fchem.2018.00368

**Published:** 2018-08-22

**Authors:** Simelys Hernández, Chiara Gionco, Thomas Husak, Micaela Castellino, José A. Muñoz-Tabares, Kristine R. Tolod, Elio Giamello, Maria C. Paganini, Nunzio Russo

**Affiliations:** ^1^CREST Group, Department of Applied Science and Technology (DISAT), Politecnico di Torino, Turin, Italy; ^2^Department of Chemistry, University of Torino, Turin, Italy; ^3^Center for Sustainable Future Technologies (CSFT), Istituto Italiano di Tecnologia, Turin, Italy; ^4^Ecole Doctorale de Chimie, Université Claude Bernard Lyon 1, Lyon, France

**Keywords:** ZrO_2_, rare-earth dopants, water splitting, third-generation photocatalysts, O_2_ evolution photocatalyst

## Abstract

In the present work, the activity of Ce and Er-doped ZrO_2_ nanopowders for sun-driven photocatalytic water oxidation has been investigated. ZrO_2_ powders with tunable amounts of tetragonal, monoclinic and cubic polymorphs have been synthesized by introducing Ce and Er (from 0.5 to 10 mol % on an oxide basis) through hydrothermal method. The aim of this work is to investigate the role of rare earth (RE) ions rich of electrons (Er^3+^) and with entirely empty levels (Ce^4+^) in the ZrO_2_ matrix for the sun-driven photocatalytic water oxidation reaction. The samples have been characterized by means of UV-Vis spectroscopy, X-Ray diffraction (XRD), N_2_ adsorption, X-ray photoelectron spectrophotometry (XPS) and transmission electronic microscopy (TEM) with energy dispersive spectroscopy (EDS). With respect to the bare ZrO_2_ mainly containing monoclinic (m-) phase, an increasing amount of rare-earth (RE) dopant was found to improve the specific BET surface area and to stabilize the tetragonal (t-) or cubic (c-) polymorphs of ZrO_2_ at room temperature. XRD data confirmed that dopants were mainly inserted in the t-ZrO_2_ phase. The photocatalytic O_2_ evolution from water under AM 1.5 G simulated sunlight illumination of the prepared samples have been correlated with their optical, structural and chemical properties. The effect of the dopant concentration on the chemical-physical and photocatalytic properties of the Er- and Ce-doped ZrO_2_ materials was elucidated. The samples with 5% of RE oxide were the most active, i.e., three times more than pure zirconia. Their superior photocatalytic activity was found to be mainly correlated to two factors: (i) an optimal surface concentration of RE ions of about 3.7%, which increased charge carriers separation in the photocatalysts surface due more superficial defects of the t-ZrO_2_ and a higher surface area, thus enhancing the reaction kinetics, (ii) a controlled amount of monoclinic vs. tetragonal (or cubic) polymorphs of zirconia with an optimum ratio of about 70/30 of t-ZrO_2_/m-ZrO_2_. Instead, the increased ability of the RE-doped ZrO_2_ to harvest visible light was found to have a secondary role on the photocatalytic activity of the Ce-doped ZrO_2_ material.

## Introduction

It is nowadays a scientific goal to develop clean energy technologies that satisfy the growing energetic demand and reduce the economic and environmental impacts of the use of fossil fuels at a global scale. Since solar radiation on the earth's surface has the potential to provide 2,850 times the current global energy needs (Ellabban et al., 2014), it pushes the idea of improving solar-driven systems for energy production and storage.

Several efforts have been done in the research community to enhance the performance of photocatalytic systems that are able to exploit solar-light irradiation for industrial applications such as pollutant abatement (Suib, 2013) and solar fuels production (Maeda and Domen, 2010). The latter process can be achieved by photocatalytic water splitting with the production of O_2_ and H_2_, or other C-containing fuels if the reducing power is used to directly reduce CO_2_. With respect to the hydrogen evolution half-reaction, water oxidation to O_2_ is more challenging because it is an up-hill reaction that involves multiple proton-coupled electron transfer processes (Armandi et al., 2013) and is considered as the bottleneck of the whole water splitting process.

The fundamental characteristic of a photoactive material (e.g., metal oxides or semiconductor photocatalysts) is that it can generate electric potential by using light as energy source. To photoexcite a photocatalyst and produce electron-hole pairs with enough oxidation and reduction potentials, the absorbed photon energy (*hv*) must be higher than its band gap energy (*E*_*g*_). Hence, the so-called first generation photoactive materials have been metal oxides or semiconductors with large band gaps and favorable flat band potentials for photocatalytic reactions, e.g., TiO_2_. However, most of them absorb only high-energy photons, i.e., UV light, which limits the utilization of solar energy to about 4%. On the other hand, semiconductors with a narrow band gap, compatible with visible light absorption, often may not generate sufficient potential for oxidation and reduction reactions.

One approach used in a second generation photoactive materials to overcome these limitations was tuning the band gap energy of semiconductors, mainly TiO_2_, by cation or anion doping in order to increase their visible light absorption by creating intra-band gap states near the top of the valence band or the bottom of the conduction band (Emeline et al., 2012). However, in most of the cases paradoxical consequences have been observed: (a) reduction of the photoactivity by extension of absorbance to the visible spectral region; (b) decrease of chemical activity of surface-active centers formed under visible light in comparison to those formed under UV illumination (Emeline et al., 2012). There are some examples in which anatase and rutile phases of TiO_2_ doped with metals such as Bi (Naik et al., 2011) and Mo (Li et al., 2011) have reported improved activity for photocatalytic water splitting under visible light irradiation by up to three times, but low productivities for H_2_ evolution have been reached.

A way to produce a next generation of photoactive materials is through band gap engineering of semiconductors with a high band gap, leading to the formation of new mid-gap states, either empty or partially populated. In that way, electron photoexcitation by low energy photons is promoted, but it is required that charges recombination involving such dopant band states have to be negligible (Emeline et al., 2012). To satisfy this condition, the energy difference between intrinsic bands and dopant band positions must be larger than 2 eV to efficiently avoid band-to-band non- radiative recombination. Modification of TiO_2_ (*E*_*g*_ ≈ 3.2 eV) often cannot guarantee an optimum photochemical response; therefore, wider band gap metal oxides like ZrO_2_ (*E*_*g*_ ≈ 5 eV) should be considered as an appropriate matrix to be modified for developing this new kind of visible light active photocatalysts (Emeline et al., 2012).

In the past decade, attention has been paid to the development of third generation visible-light-active photocatalysts based on ZrO_2_ due to its suitable flat band potentials for many reduction and oxidation reactions, low cost and high thermal and chemical stability (Ni et al., 2007). For instance, rare earth metals [e.g., Ce, (Gnanamoorthi et al., 2015), Er, Yb (Meza et al., 2010), Y, Eu, Sm and Tb (Bugrov et al., 2016)] have been employed to modify the microstructural and optical properties of ZrO_2_. Moreover, ZrO_2_ powders doped with metals, i.e. Ce and Er (Gionco et al., 2016), and non-metals, i.e. C (Poungchan et al., 2016) and N (Sudrajat et al., 2016), as well as ZrO_2_/graphene composites (Rani et al., 2016), have demonstrated photocatalytic activity for dyes degradation (such as methylene blue or rhodamine B) under visible light irradiation. Sun driven photocatalytic water splitting for direct H_2_ production from aqueous solutions, of methanol or isopropyl alcohol, has also been proved by using oxygen-deficient black ZrO_2−x_ (Sinhamahapatra et al., 2016) and ZrO_2_-Me_2_O_3_ system (where Me = Y, Eu, Tb, Sm, Er) (Bugrov et al., 2016). However, for the best of our knowledge, there are no works in which the photocatalytic water oxidation under sunlight irradiation have been demonstrated with modified ZrO_2_ samples.

In recent works (Gionco et al., 2014, 2015), some of us have shown that the dispersion of small amounts (0.5 mol %) of Ce ions in the ZrO_2_ matrix significantly modify its optical properties, becoming photoactive in visible light (at wavelengths higher than 420 nm). Further studies (Gionco et al., 2016) have shown that not only Ce but also Er doping (up to 5 mol %) improved the photoactivity of ZrO_2_ for methylene blue removal in aqueous solution under visible light. These evidences suggested further research on rare earth-doped ZrO_2_ as a potential photocatalyst for solar-driven water splitting applications.

In the present work, the activity of RE-doped ZrO_2_ powders for sun-driven photocatalytic water oxidation has been investigated. In accordance to our previous works (Gionco et al., 2014, 2016), ZrO_2_ powders containing Ce and Er (from 0.5 to 10 mol % on a RE oxide basis) have been synthesized by hydrothermal method, with the aim of testing ions rich of electrons (Er^3+^) and with entirely empty levels (Ce^4+^). The chemical, structural and optical properties of the samples have been fully studied by means of UV-Vis spectroscopy, X-Ray diffraction (XRD), N_2_ adsorption, field emission scanning electronic microscopy (FESEM) with energy dispersive X-ray spectroscopy (EDS), transmission electronic microscopy (TEM), and X-ray photoelectron spectrophotometry (XPS). Finally, the photocatalytic results have been correlated with the physico-chemical properties of the ZrO_2_-based samples, in order to elucidate the effect of the Ce and Er dopants concentration on the performance of this third generation photocatalysts for the sun-driven water oxidation reaction in the presence of an electron acceptor.

## Experimental

### Synthesis of ZrO_2_-based powders

Pure ZrO_2_ and RE-doped ZrO_2_ samples with a molar percentage of dopant of 0.5, 1, 5, and 10%, calculated on a rare earth oxide (REO) basis, were synthesized according to a published hydrothermal procedure with few modifications (Gionco et al., 2015). All reactants were purchased from Sigma-Aldrich. No further purification treatments were done before employing the reactants.

RE-doped materials were prepared starting from a 1.0 M aqueous solution, containing ZrOCl_2_·8H_2_O and Ce(SO_4_)_2_ or Er(NO_3_)_3_·5H_2_O in stoichiometric ratio. The pH of the solution was then adjusted to 11 by adding 4.0 M NaOH aqueous solution. The resulting gel was transferred into a 125 ml Teflon-lined stainless-steel autoclave. The autoclave was filled up to 70% of the total volume and then heated in an oven at 175°C overnight. The obtained precipitates were centrifuged and washed three times with deionized water. Lastly, the samples were dried at 60°C, then grounded and calcined at 500°C for 2 h.

Each sample was labeled according to its REO (CeO_2_, Er_2_O_3_) content, by using Z for ZrO_2_, C for CeO_2_, and E for Er_2_O_3_. The numbers in the label indicates the molar percentage of REO over ZrO_2_.

### Physico–chemical characterization

Semi-quantitative chemical composition analyses of the photocatalysts were evaluated by using a field emission scanning electronic microscope (FESEM, Zeiss Merlin) equipped with an electron energy-dispersive X-ray spectroscopy (EDS, Oxford X-Act) detector. EDS data was acquired on two different spots of each sample and the results are reported considering the standard deviation of those measurements.

A PHI 5000 Versaprobe scanning X-ray photoelectron spectrometer (monochromatic Al Kα X-ray source with 1486.6 eV energy), was employed to check the superficial composition of the samples. High resolution (Pass energy: 23.5 eV) and survey spectra (Pass energy: 187.85 eV) were collected by using a beam size of 100 μm. A combination of an electron gun and an Ar ion gun was used as neutralizer system to compensate the positive charging effect during the measurement. Fitting procedure and deconvolution analyses were done by using the Multipak 9.6 dedicated software. All core level peak energies were referenced to C1s peak at 284.5 eV (C-C/C-H sp2 bonds).

Insights into the crystalline structure of the ZrO_2_ powders were obtained through X-Ray Diffraction (XRD) by using a PANalytical PW3040/60 X'Pert PRO MPD with a copper Kα radiation source (0.15418 nm) and Bragg Brentano geometry. X'Pert High-Score software was used for data handling. The diffraction patterns were processed with the Rietveld refinement method to determine the crystallite size and relative abundance of phases using the MAUD 2.2 software (Lutterotti, 2010). In addition, both morphology and crystalline structure of the ZrO_2_ samples were also characterized by means of transmission electron microscopy (TEM) made with a FEI Tecnai F20ST operating at 200 kV. For the TEM analyses, the powders were dispersed in ethanol (purity >99.8 %, Sigma-Aldrich) by sonication for 5 min and then put in a TEM grid.

Diffuse Reflectance Spectroscopy (DRS) data were recorded by using a Varian Cary 5000 spectrometer, coupled with an integration sphere for diffuse reflectance studies, using Cary win UV/scan software. A sample of PTFE with 100% reflectance was used as reference.

Specific surface area of all the samples was calculated from N_2_ adsorption measurements of 9 *p*/*p*^0^ points carried out with a Micromeritics ASAP 2020 instrument and by using the Brunauer-Emmett-Teller (BET) model. Prior to the adsorption run, all the samples were outgassed at 300°C for 3 h.

### Photocatalytic water oxidation tests

**Figure 1:**
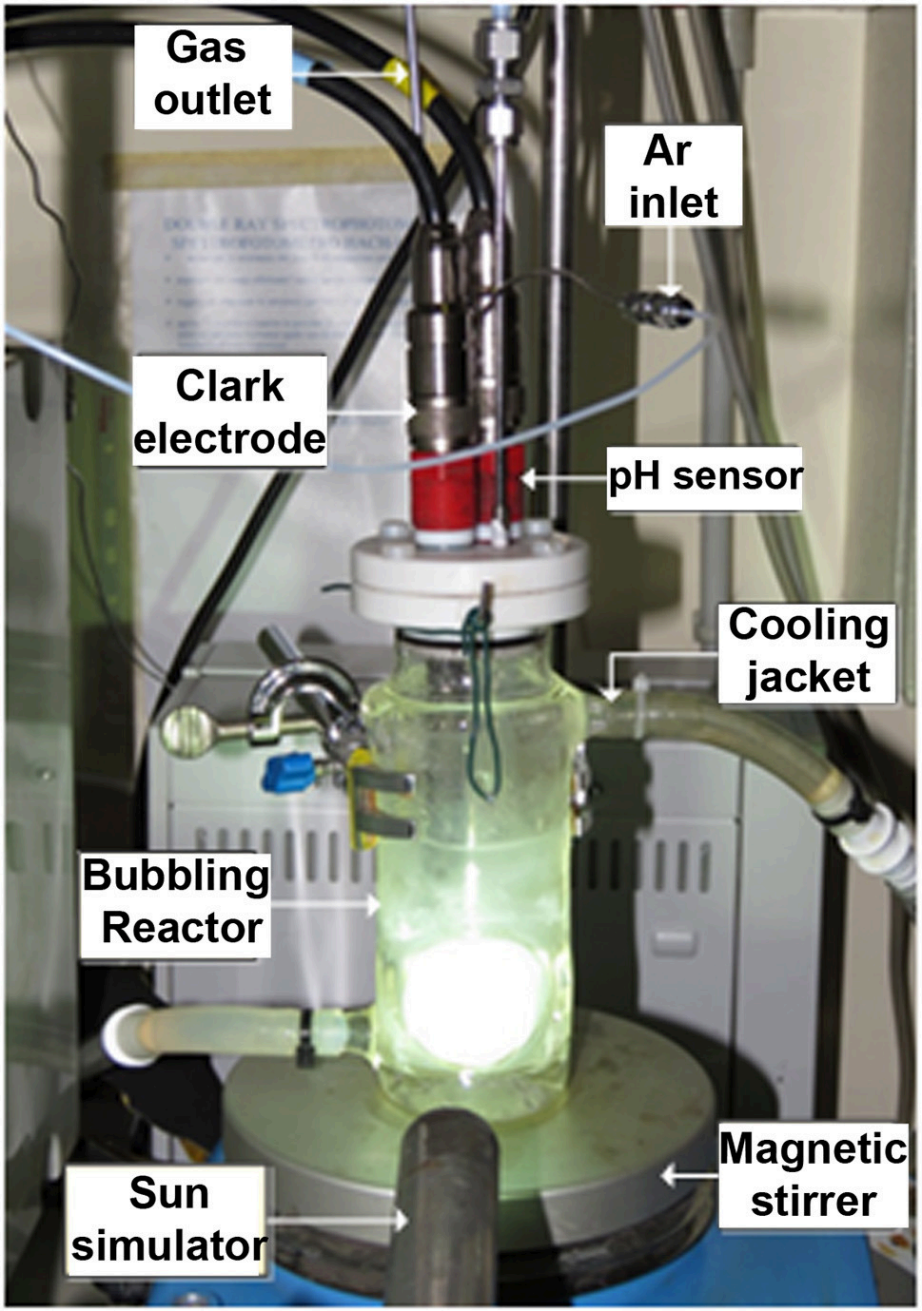
Photocatalytic bubbling reactor used for the water oxidation experiments.

A custom device was used for the photocatalytic water oxidation experiments (see Figure 1), consisting of a stirred bubbling reactor system, described in detail in previous works (Thalluri et al., 2014). In these tests, O_2_ concentration was measured online in both liquid and gas phases by means of a Clark electrode (Mettler Toledo InPro 6050/120) and a micro-Gas Chromatograph (Varian 490-GC equipped with a 10 m Molsieve 5A column and a micro-TCD), respectively. Inlet temperature and pressure were also checked, in order to maintain constant operational conditions as well as assuring the absence of leakages in the system.

The same quantity of catalyst was used for each photocatalytic test, corresponding to 100 mg of powder. The bubbling reactor was partially filled with 110 ml of AgNO_3_ 50 mM solution, which was used as electron acceptor to assess O_2_ evolution from water oxidation reaction. Then, the photocatalyst powders were dispersed in the liquid phase, thus resulting in a concentration of 0.91 g_catalyst_/l _solution_. A temperature range between 16 and 18°C was maintained in the liquid phase by using a cooling jacket. The solution pH varied between 5.5 and 7.0, depending on the tested photocatalyst. The stirring rate was set at 1,300 rpm and an overpressure of 300 mbar was kept inside the reactor during all the experiments.

At the beginning of each test, the air inside the system was purged with Ar gas up to concentrations of around 0.04 mg/l of O_2_ in the liquid phase. Then a stripping Ar was fixed at a flow rate of 18 Nml/min, by using a Bronkhorst Mass Flow Controller. A solar-light simulator with AM 1.5G spectra, model LIFI STA-40 by LUMIX (courtesy of Solaronix), was used to irradiate the reactor during a period of 70 min. The incident light irradiance was measured to be 100 mW cm^−2^ (1 sun) by using a photo-radiometer by Delta Ohm. Experimental data (dissolved O_2_, pH, temperature and pressure) were recorded by means of a LabView-based software each 4 s. Outlet gas was analyzed with the micro-GC and recorded by means of Galaxy software, which was used to register the oxygen concentrations in the gas phase each minute. The O_2_ flow produced during the reaction was calculated from micro-GC analyses every minute during the time course of the tests (see Figure S1). All the tests were repeated twice and the experimental error of the cumulative O_2_ evolved was < 10%.

## Results and discussion

### Chemical characterizations

Semi-quantitative elemental composition analyses of both the bulk and surface of the ZrO_2_ powders were obtained by means of FESEM-EDS and XPS analyses, respectively. Table 1 presents the RE atomic percentages of the samples calculated on a Zr base and theoretical values are compared to the average values obtained from both EDS and XPS. Overall, an increase in the RE currently present in the samples was observed by increasing the theoretical amount of both Ce- and Er-dopant. From bulk EDS analyses it was confirmed that almost all the samples retained similar dopant amounts to the theoretically introduced, with exception of the sample CZ10 that retained only the 60%.

**Table 1:** Chemico-physical characteristics of doped and non-doped ZrO_2_ samples theoretically calculated and obtained from XPS, FESEM-EDS, XRD, UV-Vis spectroscopy, and N_2_ absorption measurements.

**Sample**	**Theoretical REO mol % relative to ZrO_2_**	**Theoretical RE/Zr atomic %**	**Bulk RE/Zr atomic % (EDS data)**	**Superficial RE/Zr atomic % (XPS data)**	**% t-ZrO_2_/% m-ZrO_2_^a^**	**Crystals size of t-ZrO_2_ d [nm]**	**Specific surface area S_BET_ [m^2^/g]**	**Band gap Eg [eV]**
Pure ZrO_2_	0	0	0	0	34/66	7	44±2	5.15
CZ05	0.5	0.5	0.3±0.07	0.45	39/61	10	39±2	4.06
CZ1	1.0	1.0	1.0±0.35	0.40	41/59	9	44±2	3.84
CZ5	5.0	5.0	4.4±0.85	3.20	68/32	11	66±3	3.55
CZ10	10.0	10.0	6.0±0.29	11.5	75/25	12	71±4	3.41
EZ05	0.5	1.0	0.9±0.00	0.46	48/52	11	51±3	5.13
EZ1	1.0	2.0	1.7±0.03	0.80	44/56	10	63±3	5.13
EZ5	5.0	10.0	10.4±0.29	4.30	72/28	12	96±5	5.12
EZ10	10.0	20.0	17.9±2.6	6.02	98/2^b^	13^b^	88±4	5.17

a *Obtained from Rietveld refinement*.

a *c-ZrO_2_ instead of t-ZrO_2_*.

**Figure 2:**
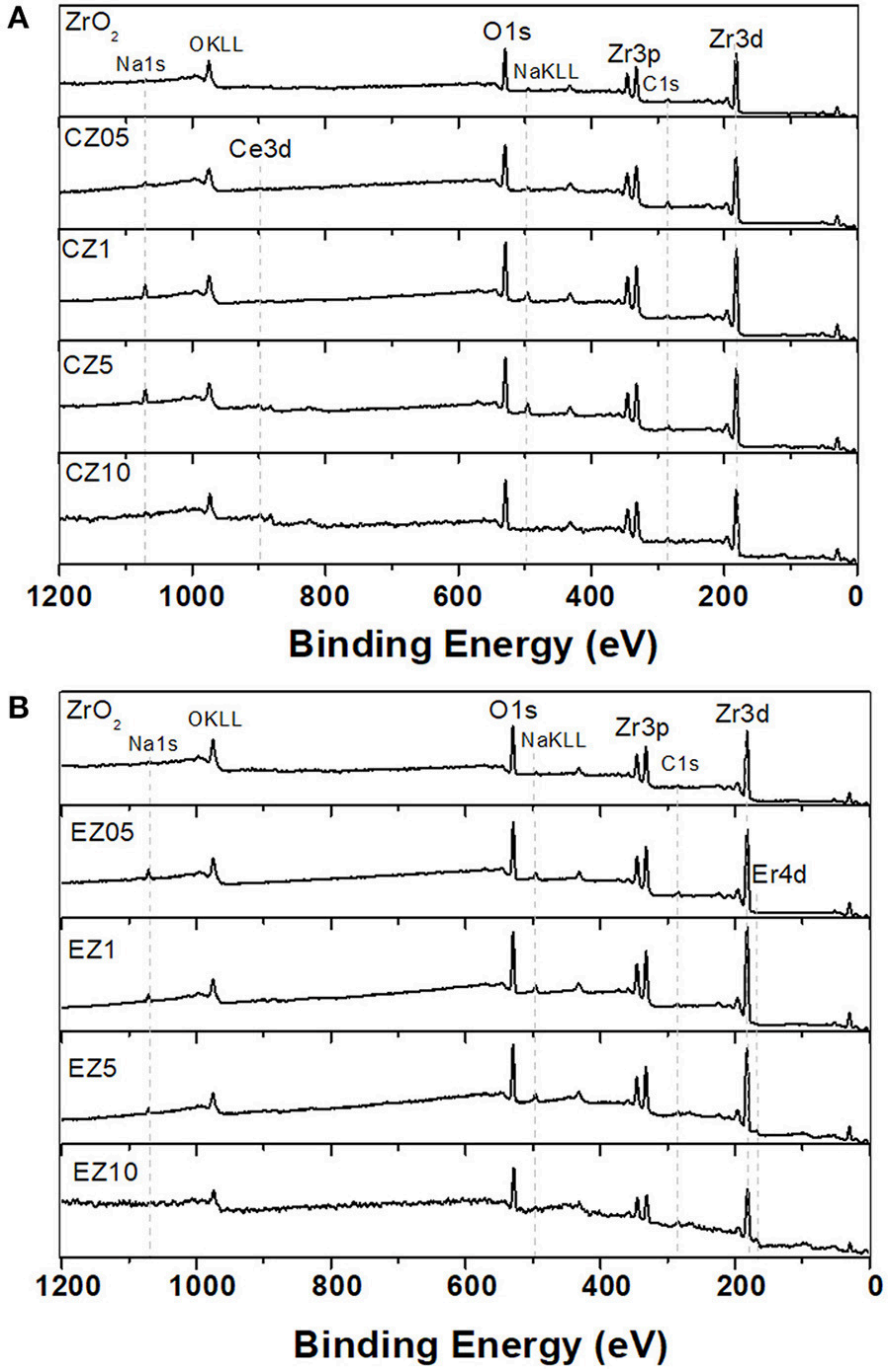
Wide-scan XPS spectra of pure ZrO_2_ and RE-doped ZrO_2_ [**(A)** RE = Ce, **(B)** RE = Er] with increasing concentration of RE dopant, as labeled in each spectrum.

The quantitative study of the XPS spectra revealed that in the Ce-doped materials, the superficial atomic percentage of Ce was close to the respective bulk values in the CZ05, CZ1 and CZ5 samples, in contrast to the CZ10 and the Er-doped ZrO_2_ samples, which evidenced about a half of the bulk RE amounts. This could be attributed to an easier distribution of Ce atoms (at the lowest percentages) than of Er during the hydrothermal synthesis process. The full-scan XPS spectrum for Ce- and Er-doped materials are presented in the (Figures 2A,B), respectively. The main peaks on the spectrum were identified Zr (Zr3p, Zr3d), O (O1s), Ce (Ce3d), and Er (Er4d). Some impurities were also found in both XPS and EDS analyses, ascribable to synthesis residues, corresponding to Na and Cl.

**Figure 3:**
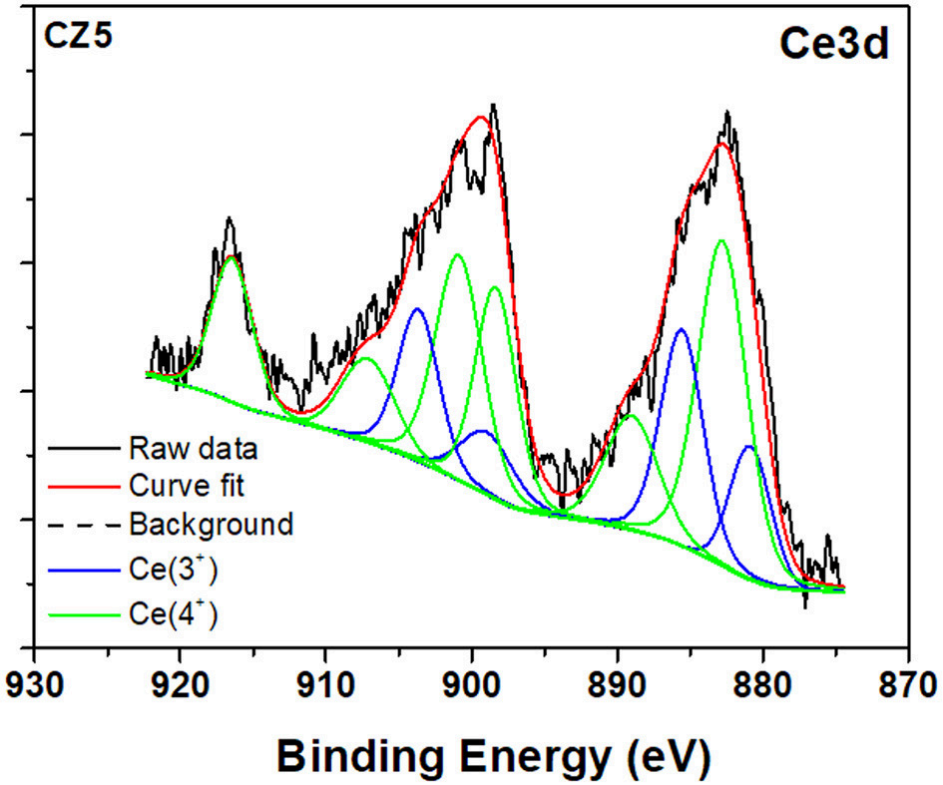
XPS high-resolution spectrum on Ce3d for 5% Ce-doped zirconia.

**Figure 4:**
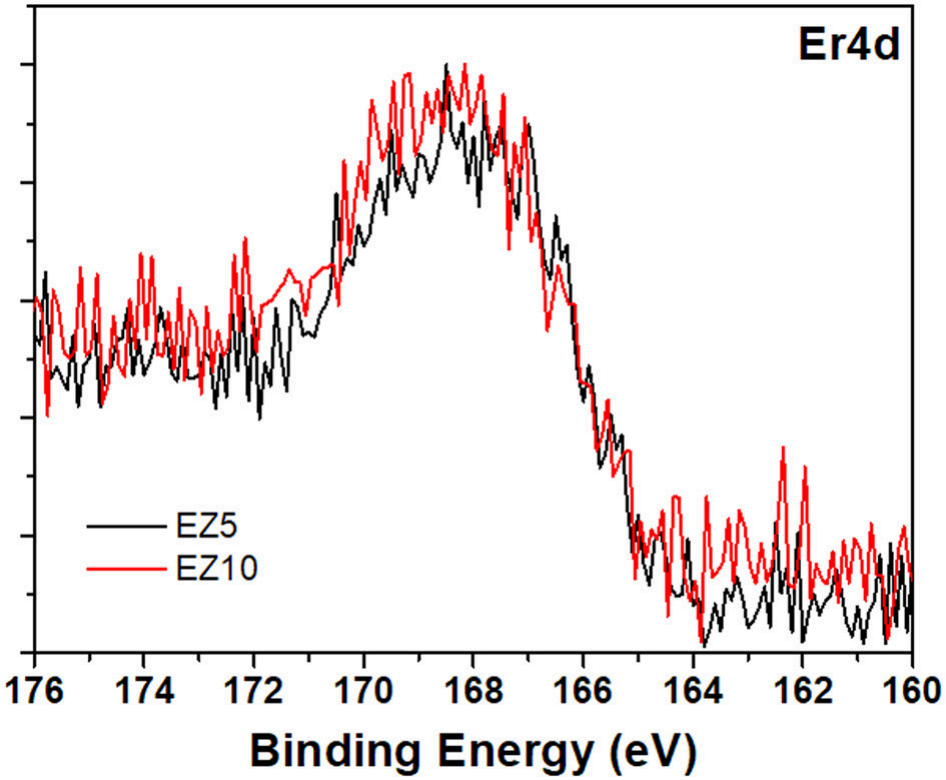
XPS high resolution spectrum on Er4d for 5 and 10% Er-doped zirconia.

Figures 3, 4 show the high-resolution (HR) XPS spectra and the curves fitting, corresponding to the Ce3d and Er4d binding energies of the samples CZ5 and EZ5, as an example. The Er4d and Ce3d peaks were examined, and identified following literature data, in order to determine the oxidation states of both dopants in the ZrO_2_ matrix. Ce3d doublets, present in all the Ce-doped samples, can be attributed to Ce^3+^ and Ce^4+^ oxidation states (Zhang et al., 2010), which confirm once again that Ce-doping actually incorporates metal ion impurities into the ZrO_2_ structure. In the case of Er4d peaks, fitting of XPS data resulted in a 168.4 eV centered peak (as shown in Figure 4), for both the EZ5H and EZ10H samples, which can be attributed to Er^3+^. The sharp peak at 167.2 eV, which should be present for Er^0^ was absent. Instead, as broad peak that is typical of a surface oxidation was observed for all the samples (Wang et al., 2015).

It is known that only from XPS data it is not possible to distinguish between Er^3+^ ions in the zirconia matrix (with oxygen vacancies as consequence of the charge imbalance) and Er_2_O_3_ in the catalyst surface (Bourbia et al., 2005). Nevertheless, XRD, TEM, and UV-Vis spectroscopy data (discussed in the next sections) were performed in order to verified the inclusion of Er ions in the Er-doped ZrO_2_ samples.

### Structural and morphological analyses

**Figure 5:**
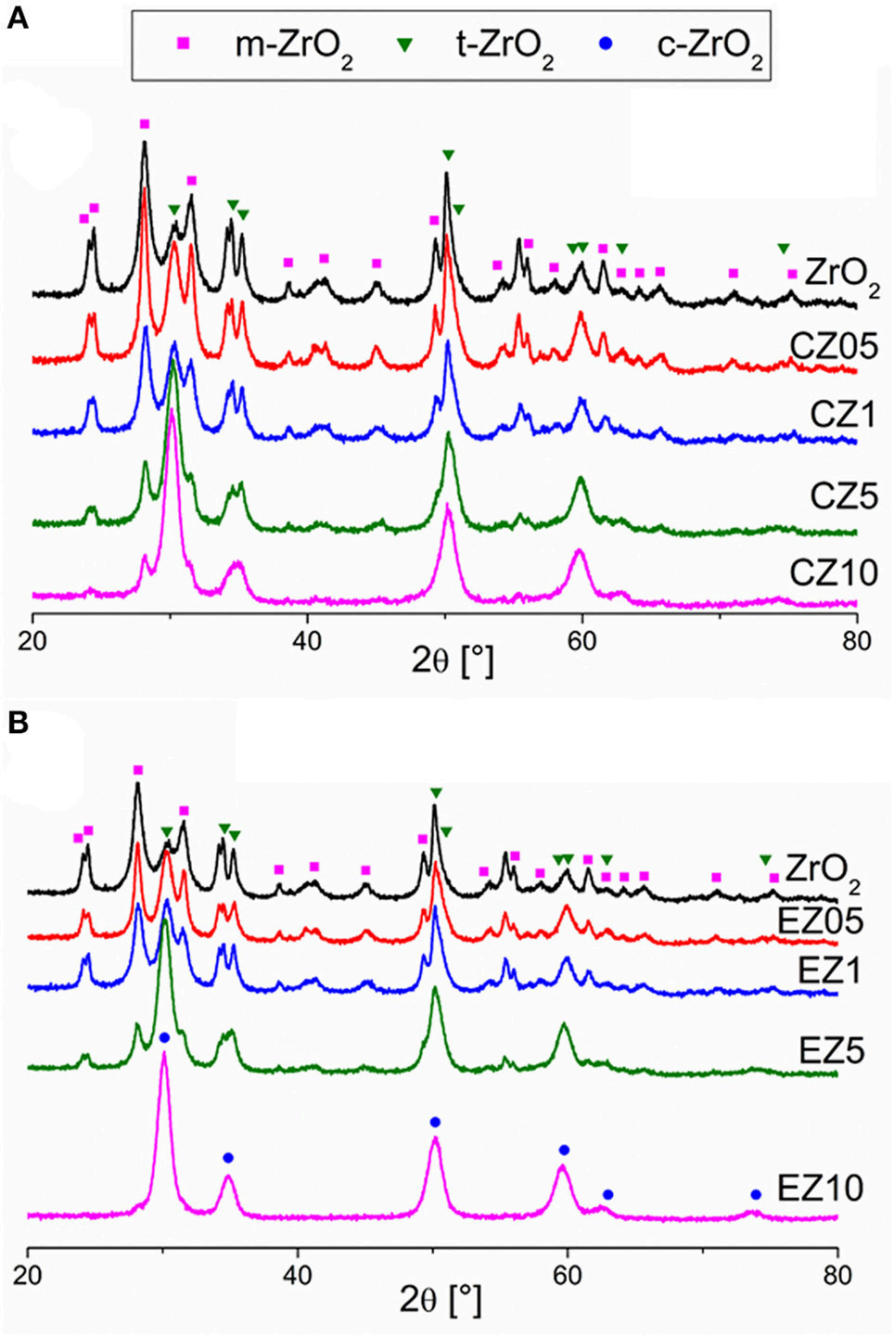
XRD patterns of pure ZrO_2_ (a, black) and RE-doped ZrO_2_ [**(A)** RE = Ce, **(B)** RE = Er] with increasing concentration of RE dopant: 0.5 mol % (b, red), 1 mol % (c, blue), 5 mol % (d, green), and 10 mol % (e, magenta).

Figure 5 shows the XRD patterns obtained for the pure zirconia, the Ce-doped (Figure 5A) and Er-doped (Figure 5B) ZrO_2_ samples. The hydrothermally synthetized ZrO_2_ samples showed a crystalline structure, with two polymorph variations; the tetragonal and the monoclinic phase. As shown in Table 1, XRD measurements evidenced that an increase in the RE-dopant content favored the formation of the tetragonal phase (t-ZrO_2_) over the monoclinic phase (m-ZrO_2_): the pure ZrO_2_ sample mainly contains monoclinic phase but, by increasing the amount of RE-dopant, the predominant ZrO_2_ phase becomes the tetragonal one. In the case of the EZ10 sample the insertion of Er probably bring to the stabilization of the cubic polymorph of ZrO_2_, as reported in literature (Jørgensen and Rittershaus, 1967). Indeed, the intensity ratio between the peaks at around 50° and around 35° has been calculated for the samples containing 5% and 10% of dopants (the other samples contain too much m-ZrO_2_ whose peaks overlap with those of t-ZrO_2_ and c-ZrO_2_ and, thus, a reliable analysis is not possible). Such ratio for c-ZrO_2_ is lower (around 1.8, ICDD Ref. code 00-049-1642) than for t-ZrO_2_ (around 2.5, ICSD Ref. code 01-079-1770). The ratio of CZ5 (2.00), CZ10 (2.23) and EZ5 (2.04) suggest the presence of t-ZrO_2_ probably with a tiny amount of c-ZrO_2_ that lowers the ratio respect to the expected one, while the ratio found for EZ10 (1.79) suggests the presence of c-ZrO_2_. Therefore, the Rietveld refinement analysis for this sample (EZ10) was performed using the latter phase instead of the tetragonal one. Crystals size of t-ZrO_2_ slightly increased (up to about 6 nm) with the introduction of both Ce- and Er-dopants. None of the samples shows the presence of diffraction peaks amenable to the formation of REO phases, indicating that likely the dopants (Er included) were successfully inserted in the ZrO_2_ matrix. Moreover, Figure S1 shows the enlargement of XRD patterns in the 26–34 2θ range. Figure S1 shows that whilst there's no evident shift in the position of the peaks related to the monoclinic phase, there's a small shift in the position of the 100% intensity peak of the tetragonal (or cubic) phase, especially for the higher dopant contents. This indicates that the RE ions were more likely inserted in the lattice of the t-ZrO_2_ or c- ZrO_2_ phases.

**Figure 6:**
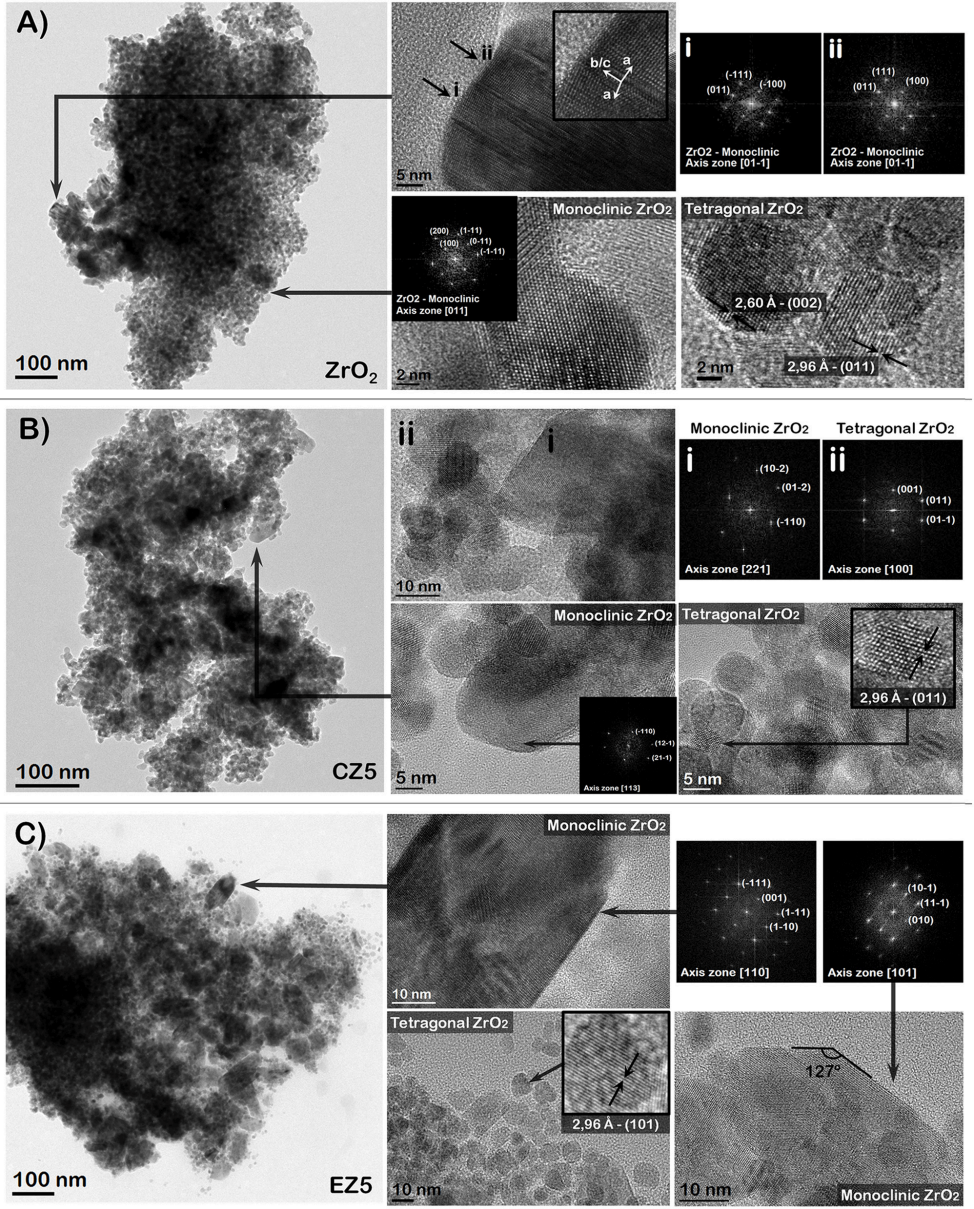
TEM (left column) and HR-TEM (center and right columns) images of pure ZrO_2_ **(A)** and RE-doped samples with 5 mol % of Ce and Er: CZ5 **(B)** and EZ5 **(C)**, respectively.

**Figure 7:**
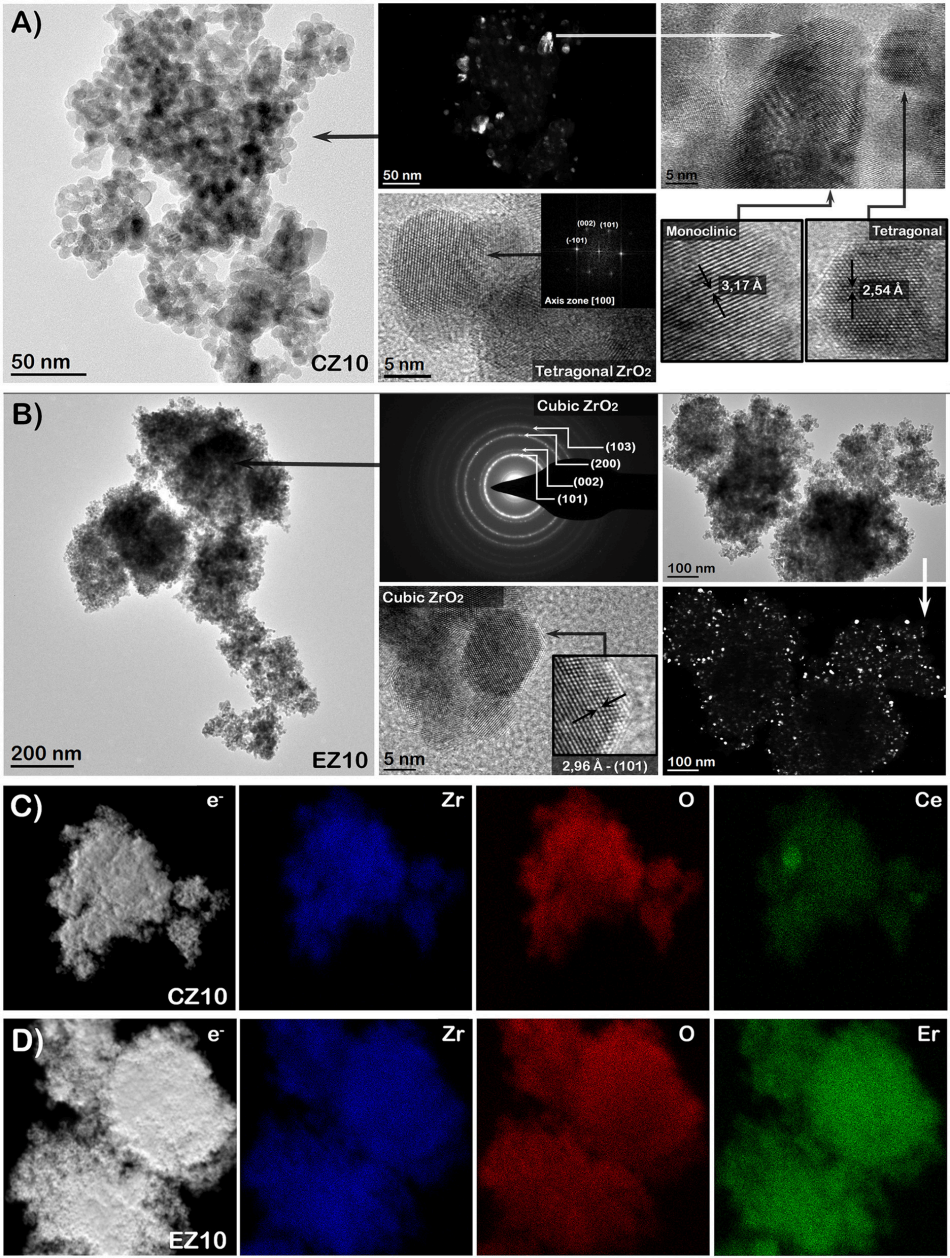
**(A,B)** TEM (left column) and HR-TEM (center and right columns) images and **(C,D)** STEM images with EDS maps of RE-doped samples with 10 mol % of Ce and Er: CZ10 **(A,C)** and EZ10 **(B,D)**, respectively.

TEM analyses were performed to further confirm the effective doping of ZrO_2_ samples and the dopants distribution. Figures 6, 7 report the TEM images of the pure ZrO_2_ and the 5 and 10 mol % Ce- and Er-doped ZrO_2_ powders, in representation of the doped samples.

From the low-magnification TEM images, two different grain morphologies could be distinguished, in both doped and non-doped samples. These are, a population of homogeneous nanocrystallites with 10 nm in diameter approximately and grains of < 100 nm in diameter. These two sorts of grains were analyzed in deep by high-resolution TEM (HRTEM). For pure ZrO_2_, it was found that 10 nm grains correspond to a mix of nanocrystallites of both tetragonal and monoclinic ZrO_2_ (Figure 6A). On the other hand, it was found that bigger grains correspond only to monoclinic phase, where many of these presented the characteristic twins (i and ii in Figure 6A) produced by the martensitic transformation tetragonal-monoclinic.

In general, the pure ZrO_2_ and most of the RE-doped samples evidenced the same kind of morphologies and crystalline phases at the TEM: (a) a mix of nanometric monoclinic and tetragonal grains of about 10 nm (similar to pure ZrO_2_ and RE-doped samples at 5%); (b) twinned grains corresponding to monoclinic phase, with a relatively larger dimension (from 15 to 60 nm), (c) bigger crystals (of about 100 nm) of only monoclinic phase. The only exception was for the EZ10 sample that was mainly formed by cubic ZrO_2_ nanocrystals with a size lower than 10 nm (in accordance to XRD data).

By increasing both Ce and Er dopants amounts to 5 and 10 mol %, the tetragonal (or cubic in the case of the EZ10 sample) phase crystals evidently increased in number with respect to the monoclinic ones (in concordance with XRD analysis). In addition, in the samples CZ5, EZ5, and CZ10, the presence of large flat monoclinic grains (Figures 6B,C, 7A) indicates that they were nucleated as monoclinic phase and then grew up, being not the product of the martensitic tetragonal-monoclinic transformation. This result is worth of notice since, in previous works, the synthesis of RE-modified ZrO_2_ samples (i.e., Y_2_O_3_-ZrO_2_) (Bugrov et al., 2016) with similar dopant contents have led to the stabilization of the tetragonal phase. Instead, the here reported hydrothermal synthesis method allowed to tune the amount of tetragonal phase vs. the monoclinic one, by varying the Ce or Er dopant content. Besides, it is noteworthy that, due to the use of Er-dopant at a concentration of 10 mol % (theoretical value based on Er oxide content), it was possible to stabilize the cubic phase of ZrO_2_ at room-temperature, while it is usually obtained at much higher temperatures (>1,000°C).

Finally, no other phases different than zirconia were found by TEM analyses. Hence, in agreement with the XRD and XPS results, no segregation of Ce or Er oxide phases were evidenced at any of the dopant concentrations, neither at the highest dopant level as shown in Figures 7A,B. This was further confirmed by STEM-EDS maps performed for all the samples. For instance, the EDS maps of the 10 mol % RE-doped ZrO_2_ samples are shown in the Figures 7C,D, where a uniform distribution of both Ce and Er dopants in the ZrO_2_ matrix can be observed, confirming that the dopants are present in all the crystalline phases of the ZrO_2_ constituting the samples.

In general, the S_BET_ in the RE-doped samples increased with respect to the pure ZrO_2_ (see Table 1). This can be ascribed to the rise in the amount of tetragonal (or cubic) ZrO_2_ phase crystals (smaller than the monoclinic ones) present in the doped materials, as it was confirmed by XRD and TEM analyses, which resulted in a reduction of the average value of ZrO_2_ crystals size. Indeed, the specific surface area (S_BET_) of bare ZrO_2_ and the low content (0.5 and 1%) Ce-doped samples was similar, while it increased in about 50 and 60% for the CZ5 and CZ10 materials, respectively. In addition, all Er doped samples presented higher S_BET_ values than bare ZrO_2_: for instance, it was enhanced in 16, 43, and 120% in the Er-doped samples with 0.5, 1.0, and 5%, respectively. All those results are in accordance to the increase of the proportion of tetragonal phase. Instead, the sample containing 10% of Er-dopant showed a slightly lower S_BET_ than the EZ5 sample that can be ascribed to the slightly bigger cubic crystals constituting the EZ10 sample.

### Optical characterization

**Figure 8:**
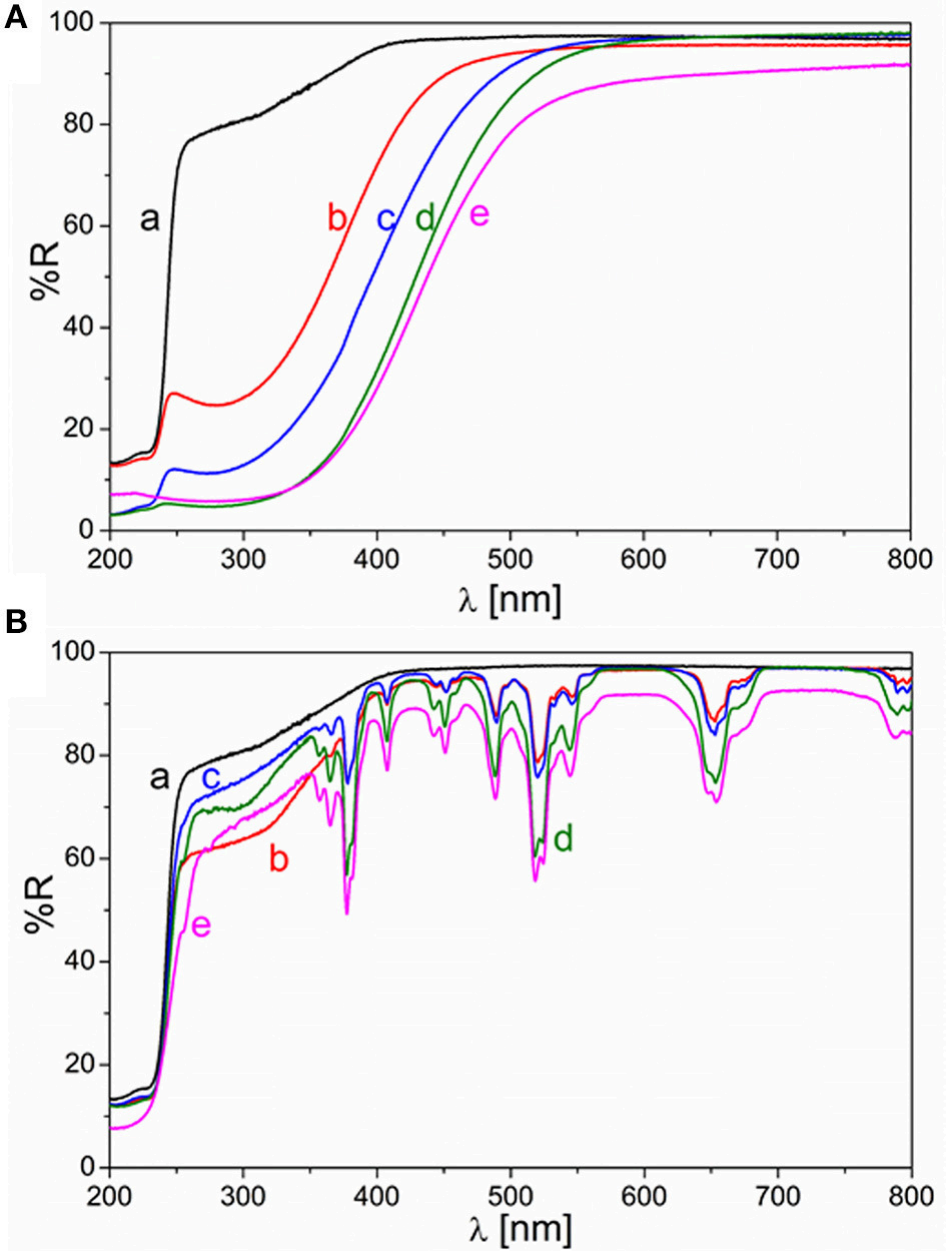
Diffuse reflectance absorbance spectra of pure ZrO_2_ (a, black) and RE-doped ZrO_2_ [**(A)** RE = Ce, **(B)** RE = Er] with increasing concentration of RE dopant: 0.5 mol % (b, red), 1 mol % (c, blue), 5 mol % (d, green), and 10 mol % (e, magenta).

Diffuse Reflectance UV-Vis spectroscopy proved that the addition of RE dopants into the ZrO_2_ matrix had a significant effect on the optical properties of the materials. Results can be seen in the Figure 8, where the spectra recorded for pure ZrO_2_ is compared with those of the doped materials.

Ce-doped ZrO_2_ samples showed a red shift of the absorption edge, which increased proportionally with the amount of dopant. In the case of Er-doped ZrO_2_, the absorption edge, and consequently the energy gap, was not shifted, on the other hand a series of sharp absorption bands in the visible region ascribable to the f-f transitions of the Er^3+^ ion appeared. The spectrum of pure ZrO_2_ is reported in both panels of Figure 8 for the sake of comparison.

The energy gap (*E*_*g*_) value was calculated for each sample by using the Tauc plot and linearizing *(*α*hv)*^2^ vs. *hv*, due to the direct band gap transition characteristic of ZrO_2_. The results are presented in Table 1 together with the surface area determined by the BET model. An increase in the concentration of Ce dopant from 0.5 to 10% caused a narrowing of the E_g_. In contrast, the percentage of Er in the samples have no effect on the energy bands position of the semiconductor material, since the bare ZrO_2_ energy gap value of around 5.1 eV was preserved for all the Er concentrations. These data is in agreement with previously reported UV-Vis analyses on similar samples with concentration of REO dopants up to 5% (Gionco et al., 2016).

### Photocatalytic water oxidation activity outcomes

**Figure 9:**
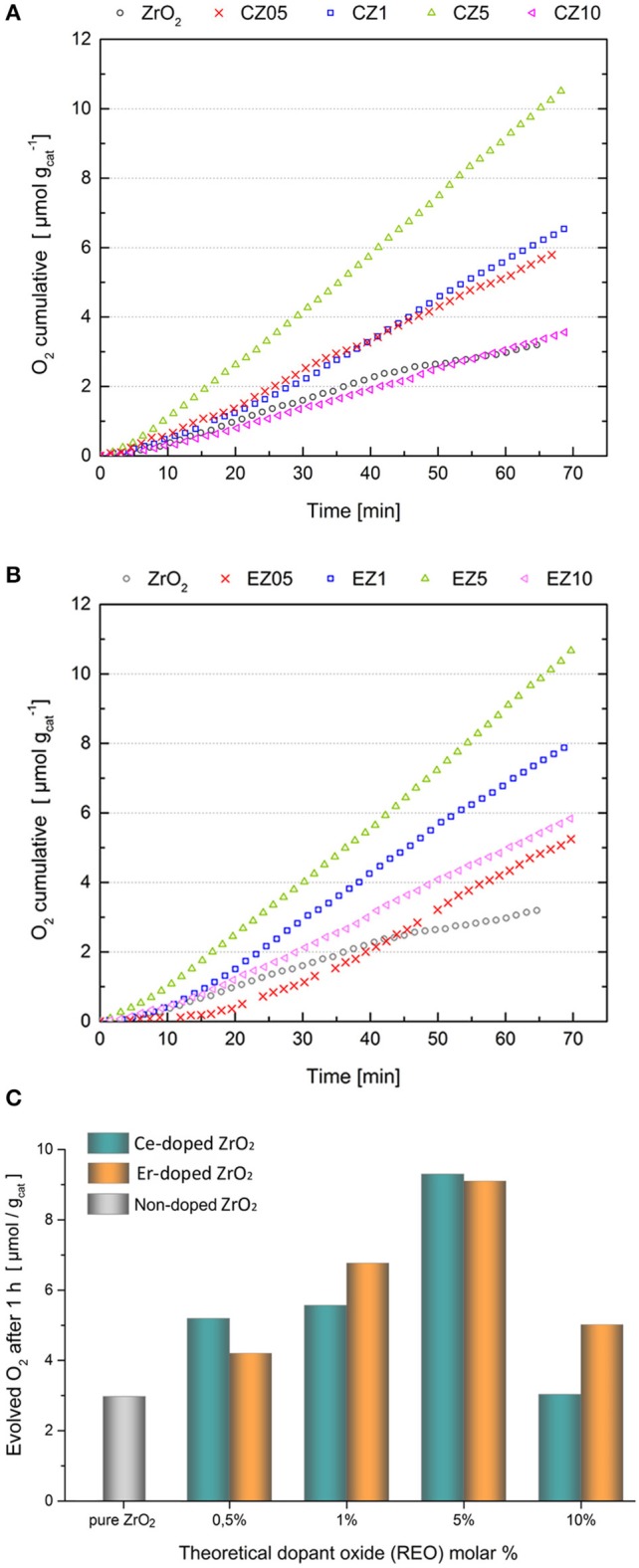
Time course of cumulative O_2_ evolved over the **(A)** Ce-doped and **(B)** Er-doped samples. **(C)** Total O_2_ evolved per gram of catalyst after 1 h of photocatalytic test of the RE-doped ZrO_2_ samples. Dopant molar percentages of 0.5, 1, 5, and 10% on a REO base. Photocatalytic test conditions: 100 mg of catalyst, 110 ml of 50 mM AgNO_3_ solution, simulated solar light irradiation (AM 1.5 G, 100 mW cm^−2^).

The performance of RE-doped and non-doped ZrO_2_ for particulate photocatalytic water oxidation (WO) in the presence of sacrificial electrons acceptor (AgNO_3_) was assessed in the bubbling reactor system as described in the section Experimental. Figure 9 reports the cumulative curves of the O_2_ evolved during the time-course of the tests and the overall O_2_ evolution measured by using the pure ZrO_2_ and Ce- and Er-doped samples, after 1 h of simulated solar light irradiation. These data were obtained by integration of the O_2_ flow produced during the reaction and measured by micro-GC analyses every minute during the time course of the tests (see Figures S2), as made in previous works of some of us (Hernández et al., 2014; Thalluri et al., 2016).

Pure hydrothermally synthetized ZrO_2_ exhibited some photocatalytic activity for solar-driven O_2_ evolution, although lower than 3 μmol h^−1^ g^−1^ catalyst, which is reasonable due to the high band gap of this material (i.e., 5 eV). In a similar way, negligible results were obtained by testing TiO_2_ Aeroxide (Degussa P25) with the same AgNO_3_ solution and simulated sunlight conditions (data not shown). Instead, the RE-doped ZrO_2_ samples evidenced a higher activity than the control ZrO_2_ and TiO_2_ samples. Both the CZ5 and EZ5 samples were the best performing photocatalysts, producing around 9 μmol g^−1^ oxygen after 1 h of irradiation, which is three times higher than that of the pure ZrO_2_. The photocatalytic activity of the Ce- and Er-doped ZrO_2_ samples was proportional to the RE content. This could be related to the enhancement of the specific surface area of these samples, in agreement with previous works on ZrO_2_ powders doped with some REO (i.e., RE = Y, Eu, Tb, Sm, Er), in which a direct correlation between S_BET_ and photocatalytic H_2_ evolution activity, during the water splitting reaction under a sacrificial electrons donor, has been observed (Bugrov et al., 2016). However, such trend was not absolute in the present case, because the performance of CZ05 and CZ1 samples was superior than such of the pure ZrO_2_ although they have similar surface area values and both CZ10 and EZ10 samples (with an enhanced S_BET_) approached the photocatalytic activity of pure ZrO_2_.

**Figure 10:**
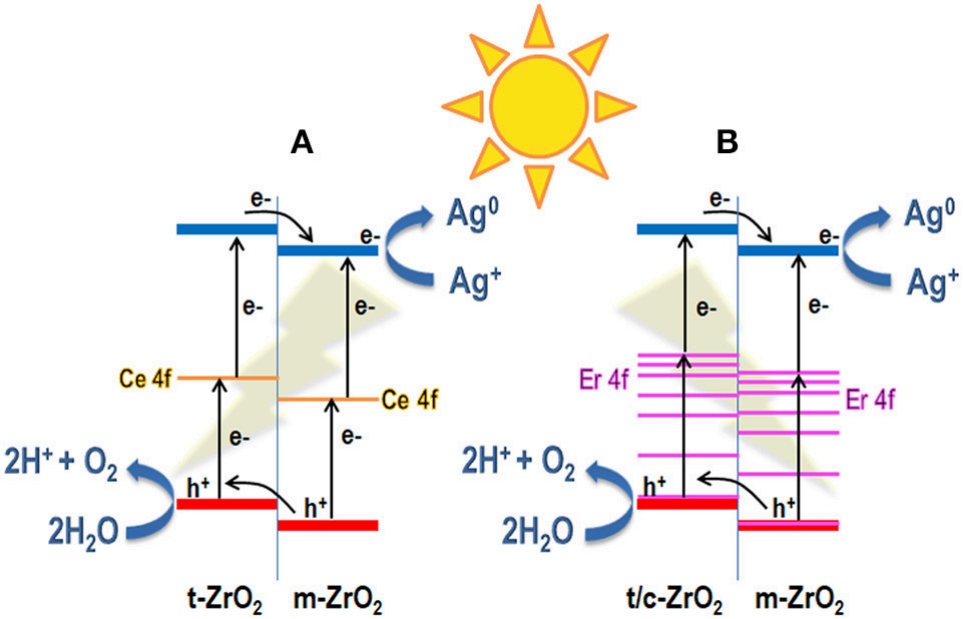
Schema of solar-driven photocatalytic water oxidation in the presence of Ag^+^ as electron scavenger under sunlight illumination over the Ce-doped ZrO_2_ **(A)** and Er-doped ZrO_2_ **(B)** samples, where it is evident the enhanced charges separation due to the presence of two ZrO_2_ polymorphs (i.e., tetragonal or cubic and monoclinic phases).

The photoactivity of the samples can be correlated to different factors and the first one is the optical properties. If the actual superficial amount of RE dopant is considered, the photocatalytic activity of the Ce-doped samples was relatively higher than that of the Er-doped ZrO_2_, up to the optimum RE ions concentration (5 mol% in REO basis). This could be explained since the Ce ions could help the absorption of visible light photons through their f states, i.e., mid gap states acting as a “ladder” (new intra band gap states that support electron transitions at lower energies), as demonstrated by DFT calculations (Gionco et al., 2014) and schematized in Figure 10A, improving the charge carriers photogeneration. Instead, in the case of the Er ions, only the f-f internal absorption bands could be exploited (see Figure 10B) acting as “antennas” for the absorption of visible light, as expected from the sharp absorption bands in the visible region of the Er-doped samples shown in Figure 8B. To confirm if the photoactivity of the ZrO_2_-doped samples is related to their visible light absorption, IPCE measurements at different wavelengths were done with the most performing RE-doped samples (i.e., CZ5 and EZ5) and on the bare ZrO_2_ deposited on FTO/glass conductive substrates (see SI for experimental details). Normalized IPCE results (shown in the Figure S3) demonstrated that, among the used RE, only the Ce-doping effectively increased the ZrO_2_ photoactivity toward the visible light region. This result agrees with the higher visible light adsorption and the lowest band gap of the Ce-doped samples than of the bare ZrO_2_ shown in the Table 1 and Figure 8A. Nevertheless, since the Er-doped samples also reported better performances than the bare ZrO_2_, the photoactivity of the here reported RE-doped ZrO_2_ cannot only be explained by an enhanced visible light adsorption, which instead seems to have a secondary role.

As a second factor, it seems evident that the water oxidation kinetics is improved in both tetragonal and cubic crystals of zirconia more than in the monoclinic ones. To confirm that, photoelectrochemical tests were performed on pure m-ZrO_2_ and t-ZrO_2_ powders deposited on FTO/glass conductive substrates (see experimental procedures in the Supporting Information). Indeed, the linear sweep voltammetries reported in the Figure S4, performed under AM 1.5G simulated sunlight illumination, demonstrated that the t-ZrO_2_ has a better photoactivity than the m-ZrO_2_ powder. This behavior could be explained by a better mobility of charges in t-ZrO_2_ than in m-ZrO_2_. However, the donor density values (calculated from Mott-Schottky plots and reported in Figure S5) of m-ZrO_2_ resulted to be higher than the one of t-ZrO_2_, which indicates that the charge transport in the bulk of the here presented t-ZrO_2_ samples is not better than such of the m-ZrO_2_ one and the same behavior was observed for the CZ5 and EZ5 samples in comparison to the bare ZrO_2_. Thus, the other possible explanation of the superior activity of the t-ZrO_2_ phase (and of the doped samples) is an improved kinetics for the water oxidation reaction due more defects on its surface and its higher surface area.

To check this possibility, XPS measurements have been performed on both pure m-ZrO_2_ and t-ZrO_2_ samples. As reported in Figure S6A, the Zr3d doublet of t-ZrO_2_ is shifted toward lower binding energies (BE, lowest BE peak at 181.2 eV) and can be deconvoluted by only two components (for Zr3d5/2 and Zr3d3/2—not reported), which can be ascribed to non-stoichiometric ZrO_x_ (Galtayries et al., 1998). Instead, the m-ZrO_2_ shows a shift toward higher BE values (lowest BE peak at 181.6 eV) and an enlarged doublet, which can be deconvoluted by four components: two for the ZrOx phase (66.6%) and two for the ZrO_2_ one (33.4%). These results are in accordance with the hypothesis that a higher degree of defects, due to surface alteration or disorder associated with oxygen vacancies, can be found on the t-ZrO_2_ surface. We also analyzed the valence band region of those samples by XPS, in order to calculate the valence band maximum (VBM) value, which is correlated with the superficial electronic conduction of the material. As reported in Figure S6B, the two VBM values (extrapolated by a linear fit in the descending side of the curve toward the Fermi Level at 0 eV) are: 1.53 eV for t-ZrO_2_ and 1.72 eV for m-ZrO_2_, which also confirm the relative positions of the valence bands reported in the Figure 10. This difference in the VBM, even if not so huge, represents a hint that the t-ZrO_2_ is more conductive on its surface, electronically speaking, than the monoclinic one, as reported by Dash et al. (2004) This behavior has also been observed in theoretical studies in which the electronic structure and density of states (DOS) of ZrO_2_ polymorphs have been investigated by DFT or LCAO calculations (Zandiehnadem et al., 1988; Vempati et al., 2015; Sinhamahapatra et al., 2016). The main result is that both materials possess a flat valence band, which is in accordance with the semiconducting behavior, however, the t-ZrO_2_ polymorph presents a higher amount of unoccupied states than m-ZrO_2_ (at around 5 eV above the Fermi level) and the O deficiency creates new available states in the gap region (Dash et al., 2004).

To further elucidate differences in the charge transfer properties of the two ZrO_2_ polymorphs, electrochemical impedance spectroscopy (EIS) measurements were performed with their photoelectrodes under simulated sunlight irradiation. Indeed, Nyquist plots of EIS shown in the Figure S7 evidence lower impedance values for the t-ZrO_2_ electrode than for the m-ZrO_2_ photoelectrode in the low frequency region (between 1 and 0.1 Hz), which correspond to slow processes happening at the electrode surface (Hernández et al., 2015, 2016) and confirm an improved charge transfer (i.e., kinetics of reaction) of the tetragonal phase during water oxidation.

From these results it is evident that both the presence of t-ZrO_2_ and the surface composition of the ZrO_2_-doped samples plays a fundamental role on their superior photocatalytic activity. Indeed, XRD data demonstrated that the dopants are mainly present in the tetragonal phase and it has been reported (Thalluri et al., 2016) that the superficial quantity of dopants plays a more important role than their total bulk amount for the water oxidation activity of visible light active powder photocatalysts, e.g. BiVO_4_. Up to a certain amount, dopants can enhance the charge carriers (e^−^/h^+^) separation and transfer from the water (e^−^ donor) to the catalyst surface and from the latter to the e^−^ acceptor (AgNO_3_). According to the photocatalytic tests (Figure 9C), the optimal dopant concentration for the here reported samples, lies around 3.7% of RE/Zr atomic ratio (i.e. the mean value of Er and Ce superficial amounts measured by XPS analyses on the CZ5 and EZ5 samples, see Table 1). However, an excess amount of Er^+3^ or Ce^+3^/Ce^+4^ also could induces the formation of a higher number of oxygen vacancies that compensate the charges in the ZrO_2_ matrix, which can also cause a higher recombination rate. Therefore, when the optimal concentration is exceeded, the dopant ions or oxygen vacancies probably acts as recombination centers, dramatically lowering the photoactivity.

This last consideration could explain the low activity demonstrated by the samples containing the highest amount of dopants (CZ10 and EZ10), which are mainly constituted by tetragonal or cubic phase of ZrO_2_, respectively. However, it also can be explained by an optimum ratio between the tetragonal and monoclinic ZrO_2_ phases of about 2.3 (mean value of the CZ5 and EZ5 samples, see Table 1). Hence, another effect that could influence the photocatalytic activity of the here reported RE-doped ZrO_2_ samples could be an enhanced charges separation at the interface between two zirconia polymorphs, monoclinic and tetragonal (or cubic) phases. This phenomenon has been observed for the water splitting reaction on commercial P25 TiO_2_ with mixed anatase and rutile crystalline phases (Masolo et al., 2015), in which the creation of a heterostructure between the two polymorphs leads on an improved interface that allow a fast separation of the photogenerated charges with a consequent reduction of recombination processes. In the present case, although the band gap values of the various phases of ZrO_2_ are quite similar (Dash et al., 2004), the VB and CB potentials of ZrO_2_ polymorphs are different, as shown in Figure 10, where a schematic representation of the relative VB and CB positions of m-ZrO_2_ vs. the t-ZrO_2_ and c-ZrO_2_ were drawn considering the VBM experimentally obtained by XPS (Figures S6) and theoretical works (Zandiehnadem et al., 1988; Hernández et al., 2015; Vempati et al., 2015).

## Conclusions

In this work, Ce- and Er- doped ZrO_2_ were successfully synthesized through hydrothermal method, with molar concentration up to 10% on a REO basis. The samples were characterized with different techniques. The addition of rare earth ions to the zirconia matrix stabilizes the tetragonal (Ce, Er) and cubic (Er, 10 mol %) polymorphs of ZrO_2_. The structural and morphological analyses point out that the rare earth ions have actually been inserted in the matrix, while the optical analysis confirms that the doping has increased the ability of zirconia of harvesting visible light.

The samples were tested for the photocatalytic water oxidation reaction by using solar simulated light in presence of a sacrificial electron acceptor. All the samples resulted active in the production of oxygen from water, with the samples containing the 5% of RE oxide being the most active (three times more than pure zirconia). We found that the highest photocatalytic activity of those samples is well correlated to an optimal amount of t-ZrO_2_ to m-ZrO_2_ phases of about 2.3 and a surface concentration of RE ions of about 3.7 mol %, which increased the charge carriers separation in the photocatalysts surface or through the interface between the monoclinic and tetragonal (or cubic) polymorphs of zirconia. Instead, the increased ability of the RE-doped ZrO_2_ to harvest visible light was found to have a secondary role in the overall photoactivity of only the Ce-doped ZrO_2_ samples.

The here presented results are of high relevance in the perspective of further optimization of a high-band gap photocatalysts as ZrO_2_ for the utilization of its high redox potential coupled to the visible light absorption for solar fuels production or other environmental applications.

## Author contributions

SH and TH tested all the photocatalysts for the sun-driven water oxidation reaction and correlated their activity to the physicochemical properties of the samples. CG synthesized the ZrO_2_ powders and performed XRD and UV-vis spectroscopy characterizations. MC did and analyzed XPS measurements. JM-T performed TEM imaging and analyses. KT did the photoelectrochemical measurements and data analysis. NR contributed with the XRD and XPS data analysis. EG, MP, and NR supervised the experimental work. All the authors contributed with discussion of results and writing of the manuscript.

### Conflict of interest statement

The authors declare that the research was conducted in the absence of any commercial or financial relationships that could be construed as a potential conflict of interest. The reviewer MR and handling Editor declared their shared affiliation.

## Supplementary material

The Supporting Information contains two sections: (i) Materials and Methods describing synthesis of pure t-ZrO_2_ and m-ZrO_2_ samples, preparation of the photoelectrodes and photoelectrochemical (PEC) tests and (ii) Figures of XRD data (magnification), example of O_2_ molar concentration calculated from continuous micro-GC analyses during the time-course of photocatalytic experiments, PEC results and XPS plots of pure t-ZrO_2_ and m-ZrO_2_ samples.
