# Supplementary material for: Insights Into the Sunlight-Driven Water Oxidation by Ce and Er-Doped ZrO2
Source: Front Chem. 2018 Aug 22;6:368. doi: 10.3389/fchem.2018.00368 (PMC6113700; doi:10.3389/fchem.2018.00368)
Supplement: Supplementary file 1 [file Data_Sheet_1.docx]

**Insights into the sunlight-driven water oxidation by Ce and Er-doped ZrO_2_**

Simelys Hernández,^a,*^ Chiara Gionco,^b,*^ Thomas Husak,^a^ Micaela Castellino,^c^ José Alejandro Muñoz-Tabares,^c^ Kristine Rodulfo Tolod,^a,d^ Elio Giamello,^b^ Maria Cristina Paganini ^b^ and Nunzio Russo ^a^

^*^ corresponding authors: [simelys.hernandez@polito.it](mailto:simelys.hernandez@polito.it); [chiara.gionco@unito.it](mailto:chiara.gionco@unito.it)

^a^ CREST group, Department of Applied Science and Technology, DISAT, Politecnico di Torino, 10129, Turin, Italy.

^b^ Department of Chemistry, University of Torino, Via Giuria 7, 10125, Turin, Italy

^c^ Center for Sustainable Future Technologies, CSFT, IIT@Polito, Istituto Italiano di Tecnologia, Corso Trento 21, 10129, Turin, Italy.

^d^ Ecole Doctorale de Chimie, Université Claude Bernard Lyon 1, 69100, Villeurbanne, Lyon, France

**Supporting Information**

**Materials and Methods**

***Synthesis of pure t-ZrO_2_ and m-ZrO_2_ samples:***

Two reference samples of pure t-ZrO_2_ and m-ZrO_2_ were prepared to perform photocatalytic measurements to elucidate the role of the different polymorphs on the water oxidation ability of ZrO_2_. To obtain the pure t-ZrO_2_ sample, a 0.5M water solution of Zr oxychloride octahydrate was prepared and then, its pH was adjusted to 10 by using a 4.0 M NaOH aqueous solution to induce a gel formation. The gel was placed in a microwave oven, heated at 800W for 1 minute and then cooled down naturally. The so obtained precipitate was centrifuged and washed three times with de-ionized water and then dried at 70 °C. The material was grinded and calcined in a muffle furnace at 600°C for 1 hour.

To obtain the pure m-ZrO_2_ sample, a 0.67M water solution of Zr oxychloride octahydrate was prepared and its pH was adjusted to 10 by using a 4.0 M NaOH aqueous solution to form a gel. The gel was then transferred into a 125 ml Teflon-lined stainless-steel autoclave, which was filled up to 70%, closed and heated in an oven at 200 °C for 24 hours. The precipitate was then centrifuged and washed three times with de-ionized water and then dried at 70 °C.

***Preparation of the photoelectrodes:***

The as-synthesized ZrO_2_ powder were deposited into electrodes via the doctor blade technique. For each of the samples, 0.2 grams of the powder was mixed with 304 μL of ultrapure H_2_O (18 MΩ.cm at 25^o^C), 31 μL of CH_3_COOH (≥ 99%, Sigma-Aldrich) and 619 μL of absolute ethanol (≥ 99.8%, Carlo Erba). The mixture was sonicated for 4 hours at room temperature to obtain a paste. The paste was then applied on the conductive side of fluorine-doped tin oxide (FTO, 7 Ω sq^-1^ by Solaronix) coated glasses via the doctor blade technique. Then, the electrode samples were calcined at 500^o^C for 2 hours to improve the adhesion of the powder on the FTO substrate.

***Photoelectrochemical (PEC) measurements:***

A three-electrodes system with a Pt coil counter electrode (CE), Ag/AgCl (3M KCl) reference electrode (RE), and the fabricated photoelectrodes as working electrode (WE), were set up in a single-compartment quartz cell containing a 0.1 M phosphate buffer solution at pH 7. PEC activity tests were performed by using a BioLogic VSP 300 potentiostat. For the conversion of the measured potentials versus the Ag/AgCl (3M KCl) reference electrode to RHE (NHE at pH=0), the Nernst equation E_RHE_=E_(Ag/AgCl)_ + 0.059pH + E_(Ag/AgCl)_^o^ (E_(Ag/AgCl)_^o^ = +0.199V ) was applied.

To evaluate the visible light activity of the doped-ZrO_2_ samples in comparison to the bae ZrO_2_, Incident photon-to-electron conversion efficiency (IPCE) spectra were recorded using a Newport Xe lamp (150 W) coupled to a monochromator (Cornestone 130 by Newport), by varying the wavelength of the incident light from 320 nm to 550 nm (step size: 10 nm), at an applied potential of 0.61 V vs. Ag/AgCl (1.23 V vs. RHE). The photoelectrode illuminated area was of 1 cm^2^ and the light power density was of about 1 mWcm^2^ (measured at 390 nm).

For characterizing the photocatalytic activity of the pure t-ZrO_2_ and m-ZrO_2_ photoelectrodes, linear sweep voltammetry (LSV) under illumination was employed in the range -0.3 V to 1 V vs. Ag/AgCl at a scan rate of 10 mV/s. The LSV curves show the behavior of the photocurrent density as a function of the applied potential in the PEC system. Charge transport and transfer properties of those photoelectrodes were studied by electrochemical impedance spectroscopy (EIS) measurements, in the frequency range of 0.1 Hz to 0.5 MHz, at 0.61 V vs. Ag/AgCl (1.23 V vs. RHE). To simulate sunlight irradiation in both LSV and EIS tests, a Newport 450 W Xe lamp with an AM 1.5G filter was used, maintaining an intensity of 100 mW/cm^2^.

To examine the carrier mobility and the photocatalytic potential of the photoelectrodes, staircase potentio-electrochemical impedance spectroscopy (SPEIS) measurements were performed under dark conditions for each sample, in the potential range of -0.8 V to 1 V (vs. Ag/AgCl), at a frequency of 7.5 kHz with an amplitude of 25 mV. Then, Mott-Schottky plots were drawn to extrapolate the flat band potentials (E_fb_) and donor densities (N_D_) of the photoanodes, in according to the equation (1):

$\frac{1}{C^{2}}=\frac{2}{{}_{o}A^{2}eN_{D}}\left( E-E_{fb}-\frac{k_{B}T}{e} \right)$ (1)

where C is the interfacial capacitance, ε is the dielectric constant of the semiconductor, ε_o_ is the permittivity of free space, A is the area of the interfacial capacitance, N_D_ is the donor density, E is the applied potential, E_fb_ is the flat-band potential, k_B_ is the Boltzmann’s constant, T is the temperature, and e is the electronic charge. A plot of (1/C^2^) A^2^ versus the potential yields a linear region. From the value of this slope, the N_D_ of a semiconductor can be calculated and the intercept of the extrapolation of this linear plot to (1/C^2^) A^2^ = 0 gives its flat band potential. A positive slope indicates that the material is an n-type semiconductor and electrons are the majority charge carriers.

**Figures**

**Fig. S1:** Enlargement in the 26-34 2θ range of XRD patterns of pure ZrO_2_ (a, black) and RE-doped ZrO_2_ (panel **A**: RE = Ce, panel **B**: RE = Er) with increasing concentration of RE dopant: 0.5 mol % (b, red), 1 mol % (c, blue), 5 mol % (d, green) and 10 mol % (e, magenta).


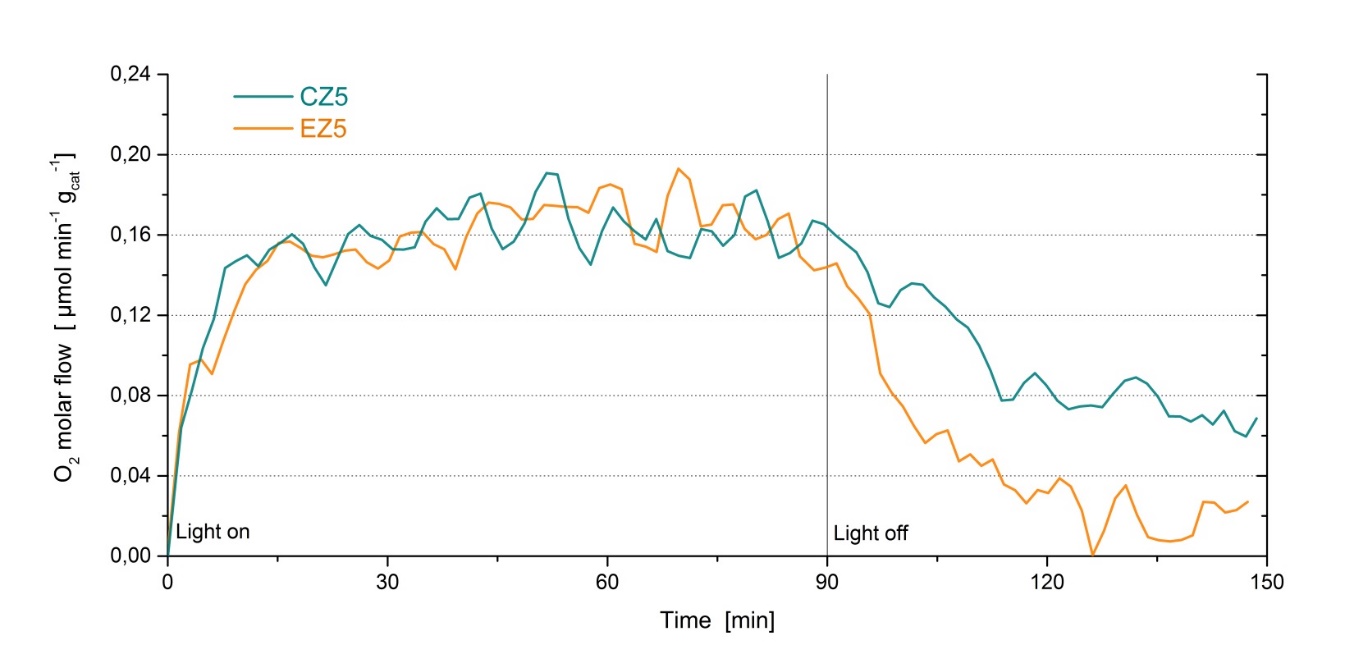


**Fig. S2**: O_2_ molar flow over time per gram of catalyst under simulated solar light irradiation (AM 1.5G, 100 mW cm^-2^) measured by using the CZ5 and EZ5 samples –hydrothermally synthetized ZrO_2_ powders with 5 % dopant molar percentages on a CeO_2_ and Er_2_O_3_ base, respectively–. A stripping Ar flow rate of 18 Nml min^-1^ was maintained during the experiments. Tests were done with 110 ml of a 50 mM AgNO_3_ solution containing 100 mg of catalyst powder. The O_2_ baseline was settled up at 0 μmol min^-1^ before starting the illumination of the samples.

**Fig. S3**: Incident-to-Photon-Current-Efficiency (IPCE) normalized by the mass (g) of catalyst representing the action spectra vs. wavelength of the CZ5 and EZ5 samples *versus* the bare ZrO_2_. Measurements made with the photoelectrodes of the powders deposited in FTO/glass substrates tested in 0.1M Na-phosphate buffer (pH=7), under simulated AM1.5G sunlight irradiation of 100 mA/cm^2^.

**Fig. S4**: (a) LSV of photoelectrodes of with t-ZrO_2_ and m-ZrO_2_ powders deposited in FTO/glass substrates, tested in 0.1M Na-phosphate buffer (pH=7), under simulated AM1.5G sunlight irradiation at 100 mA/cm^2^ at a scan rate of 10 mV/s.

| **Sample** | **E_fb_ (V vs RHE)** | **Donor density (x 10^20^cm^-3^)** |
| --- | --- | --- |
| Bare ZrO_2_ | -0.61 | 13.1 |
| Ce-ZrO_2_ | -0.55 | 9.2 |
| Er-ZrO_2_ | -0.53 | 11.5 |
| m-ZrO_2_ | -0.59 | 35.5 |
| t-ZrO_2_ | -0.53 | 18.7 |

**Fig. S5**: Mott-Schottky Plots of t-ZrO_2_ and m-ZrO_2_ obtained from SPEIS data measured at a constant frequency of 7.5 kHz under dark conditions.


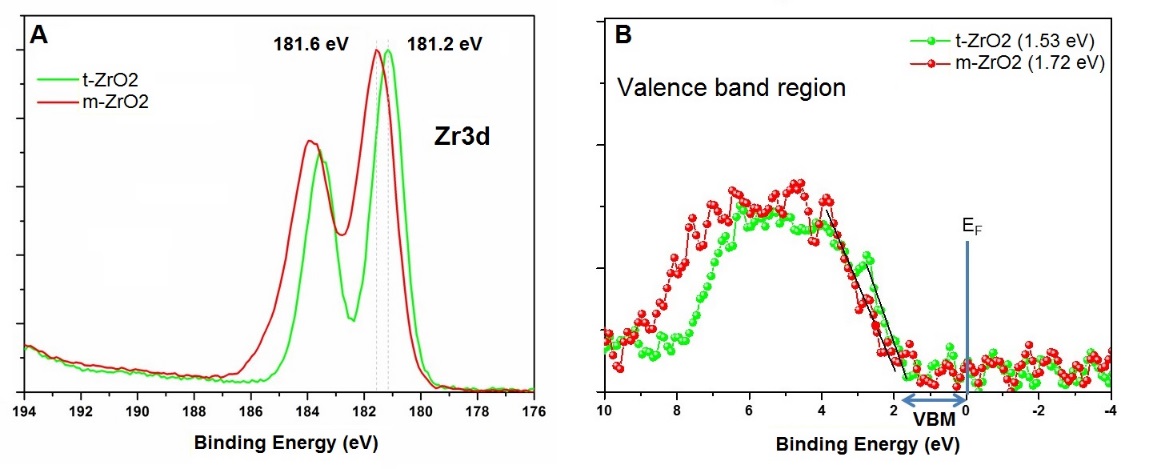


**Fig. S6**: XPS curves for t-ZrO_2_ and m-ZrO_2_ samples: A) Zr3d doublet and B) valence band region with Valence Band Maximum (VBM) values reported towards the Fermi level (E_F_).


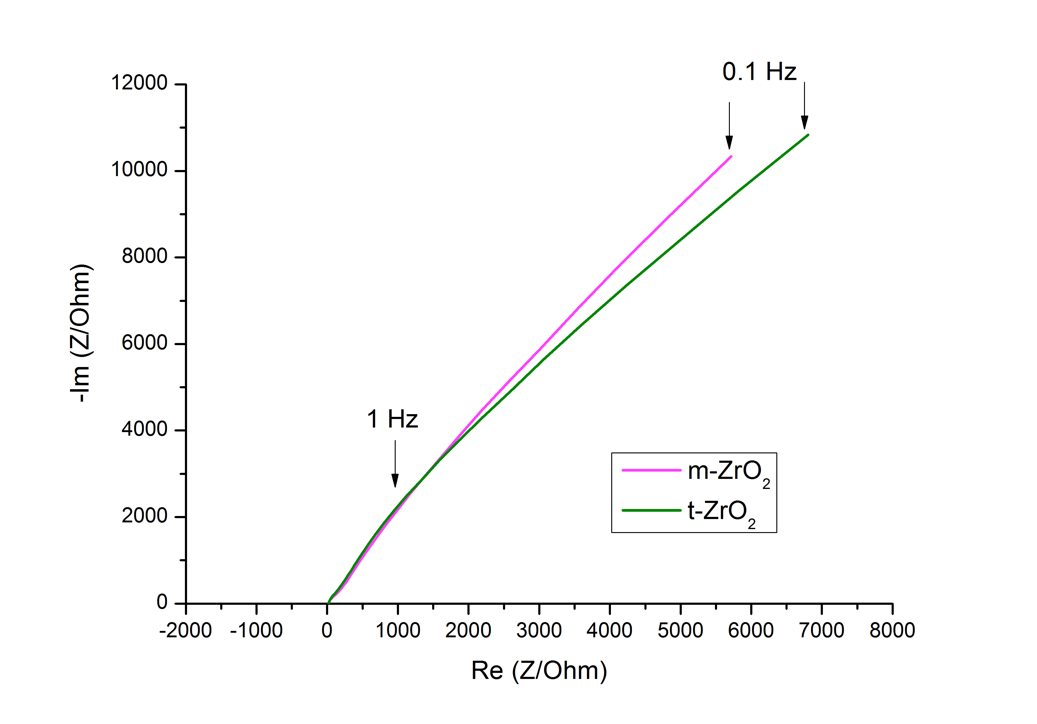


**Fig. S7**: Nyquist plots of the EIS measurements of t-ZrO_2_ and m-ZrO_2_, acquired at 1.23 V vs RHE under simulated AM1.5G sunlight irradiation (100 mW/cm^2^).
